# P-488. Factors associated with the first-time HIV testing among priority populations in Kentucky

**DOI:** 10.1093/ofid/ofae631.687

**Published:** 2025-01-29

**Authors:** Jaime Soria, Jana Collins, Amanda B Wilburn, James Thacker, Ardis Hoven, Nicholas Van Sickels, Alice C Thornton

**Affiliations:** University of Kentucky, Lexington, Kentucky; University of Kentucky, Lexington, Kentucky; The University of Kentucky, Lexington, Kentucky; University of Kentucky, Lexington, Kentucky; University of Kentucky, Lexington, Kentucky; University of Kentucky, Lexington, Kentucky; University of Kentucky, Lexington, Kentucky

## Abstract

**Background:**

The Kentucky Income Reinvestment Program (KIRP), a statewide program funded through the Ryan White HIV/AIDS Program, provides comprehensive early intervention services targeting persons at the highest risk for HIV infection. Conducting first-time HIV testing in high-risk populations represents a crucial and proactive approach to mitigating the impact of HIV/AIDS, plays a pivotal role in reducing onward transmission within communities, and provides critical data for public health planning.

**Figure:**
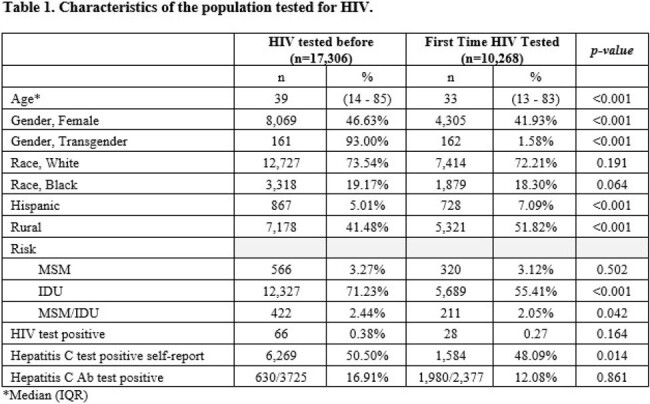

**Methods:**

We conducted a cross-sectional study in Kentucky from January 2022 to March 2024. We compared the demographic characteristics and factors associated with first-time HIV testing in high-risk populations. Study data were collected and managed using a REDCap database. The statistical analyses, including logistic regression, were performed with Stata 17 (College Station, TX: Stata Corp LLC).

**Figure:**
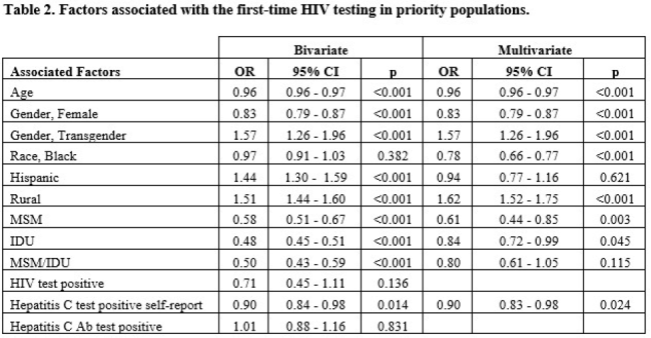

**Results:**

In the study period, 27,574 subjects were tested for HIV, and 10,268 (37.2%) of them reported not having had an HIV test before. The median age for the first-time tested was 33 (IQR 13 – 83) years; 4,305 (41.9%) were female, 162 (1.58%) were transgender, and 5,321 (51.8%) lived in Rural Counties. (Table 1) In a multivariate analysis, first-time HIV test was associated significantly with younger age, transgender population, and living in rural counties. (Table 2)

**Conclusion:**

The study underscores the critical importance of reaching high-risk populations for initial HIV testing. Around 40% of the subjects tested did not have a previous HIV test, even though they belonged to a high-risk group. The findings reveal several key demographic factors associated with first-time HIV testing, including the younger population, transgender identity, and rural residents, who were more likely to undergo their initial HIV test through the KIRP interventions to address demographic and geographic disparities.

**Disclosures:**

**All Authors**: No reported disclosures

